# Tissue origin dictates functional diversity of BMSC-derived EVs in bone regeneration

**DOI:** 10.3389/fcell.2025.1675425

**Published:** 2025-11-18

**Authors:** Jia Wang, Jinjia Hong, Chunrui Tian, Xiangyu Zhao, Qingpeng Xie, Na Li, Yuan Zhang, Meixian Liu, Pengfei Zhang, Helin Xing, Lu Wang, Xing Wang

**Affiliations:** 1 Shanxi Medical University School and Hospital of Stomatology, Taiyuan, China; 2 Shanxi Province Key Laboratory of Oral Diseases Prevention and New Materials, Taiyuan, China; 3 Department of Prosthodontics, Beijing Stomatological Hospital and School of Stomatology, Capital Medical University, Beijing, China

**Keywords:** extracellular vesicles, BMSCs, bone, bone regeneration, miRNA

## Abstract

Bone regeneration is mediated by paracrine signaling, with extracellular vesicles (EVs) playing a crucial role as communication mediators. Previous studies have shown that there are differences in the performance of the bone marrow mesenchymal stem cells (BMSCs) derived from the mandible and limbs. However, it is not clear whether there are differences in EVs originating from them. In this study, we demonstrated that compared to EVs derived from limbs, the EVs derived from the mandible were more effective in stimulating BMSCs migration, proliferation, osteogenic differentiation *in vitro*, and bone regeneration *in vivo*. Upregulated miRNAs in EVs from mandible target signaling pathways such as MAPK, Wnt, and Hippo, which have been shown to be crucial for bone formation. Therefore, it may be an excellent candidate for improving bone healing in autologous bone transplantation, bone tissue engineering, or other bone diseases.

## Graphical Abstract

**Figure:**
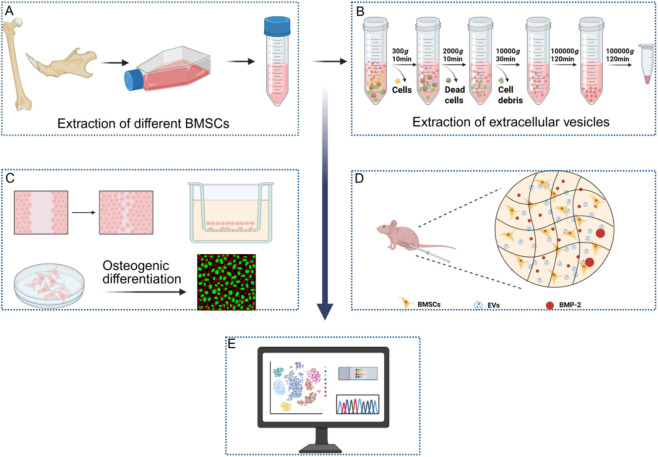
**(A)** Extraction of BMSCs. **(B)** Extraction of EVs. **(C)** Effects of EVs on proliferation, migration and osteogenic differentiation. **(D)** Effects of EVs on bone formation *in vivo*. **(E)** Transcriptomic analysis. (Created on Biorender).

## 1 Introduction

Bone regeneration is mainly regulated by paracrine signals, among which extracellular vesicles (EVs) have become a key substance (Linero and O Chaparro, 2014). Bone marrow mesenchymal stem cells (BMSCs) play an important role in this process (Wu, et al., 2024). The main problems of BMSCs in clinical application are immunogenicity and biosafety (Kai, et al., 2018; Maria Elisa et al., 2021; Meiwand, et al., 2022). MSCs-derived EVs have low immunogenicity and are more safe to use (Hojun, et al., 2022; Fei, et al., 2024; Jafar, et al., 2017). EVs carry a variety of factors, especially microRNAs (miRNAs) and long noncoding RNAs (lncRNAs), which can be internalized by target cells and affect gene expression (Jagannath, et al., 2023; Xingyu, et al., 2023; Han, et al., 2023; Kayla, et al., 2023; Teng, et al., 2018; Hou-Fu, et al., 2023).

The functionality of EVs is influenced by the origin of the donor cells (Tingting, et al., 2023; Menghui, et al., 2023; Ruoxi, et al., 2022; 家宇 李, 2024; Huan, et al., 2024; Yuanliang, et al., 2022). EVs from different tissues have different miRNA profiles, indicating that they may play a role in a tissue-specific manner. For example, adipose-derived EVs have a stronger role in promoting bone and cartilage regeneration compared EVs from synovial stem cells. In addition, different types of EVs have different effects on cell proliferation, dermal fibroblast migration, and keratinocyte formation (Diem Huong, et al., 2020).

Mandible and limb bones have different developmental origins and ossification patterns. The mandible originated from ectoderm and developed intramembranous ossification; Limb bones originated from mesoderm and formed through endochondral ossification Michael, 2021; Mirca, et al., 2015). It was found that BMSCs derived from mandible had better proliferation ability, larger colony forming units and more calcium deposition. Osteoblasts from the mandible also have a stronger ability to promote angiogenesis (Xue, et al., 2019; Xin, et al., 2020; Maria Pia, 2023). It is still unclear whether there are significant differences between these two different sources of EVs.

This study systematically investigated the differences in EVs derived from mandible and limb BMSCs. Through the study of physiological function *in vitro* and bone regeneration model *in vivo*, it is found that EVs from mandible has stronger function of promoting osteogenesis and bone regeneration, which provides some scientific guidance for clinical application.

## 2 Materials and methods

### 2.1 BMSCs culture and identification

The muscles and periosteum of mandible and limb bones of SD rats (6–8 weeks old, male, 120–140 g, n = 18) were dissected. The bone block was rinsed with phosphate buffered saline (PBS), and the bone marrow cavity was rinsed with 1 mL syringe to collect BMSCs. For the mandible, the mandibular ramus, alveolar bone and teeth were removed, and the remaining mandible was shredded in sterile PBS to allow bone marrow to flow out. After centrifugation of the bone marrow washing solution, the supernatant was discarded, and the cells were resuspended in complete growth medium and cultured. The medium consisted of 10% fetal bovine serum (FBS) and 1% penicillin/streptomycin in MEM-α. The cells were digested and placed in a staining buffer containing fluorescent labeled antibodies against CD44, CD73, CD90, CD105, CD34 and CD45. After incubating in dark for 20 min, the cells were washed twice with PBS and resuspended for flow cytometry. This research scheme has been approved by the ethics committee of Shanxi Medical University.

### 2.2 EVs isolation and purging

The BMSCs were cultured in 175 cm^2^ culture flasks (Nest, China) until they reached 90% confluence. After washing twice with PBS (Servicebio, China), cells were cultured in the medium containing 10% exosome-depleted serum. The exosome-depleted serum was acquired through ultracentrifugation at 150,000 × g for 12 h at 4 °C. After incubating for 48 h, the supernatant was gathered, and differential centrifugation was employed to isolate EVs.

In brief, the cell-free supernatant underwent centrifugation at 300 *g* for 10 min to remove cells. Subsequently, centrifugation at 2,000 × g for 10 min and 10,000 × g for 30 min was performed to discard cell debris and large vesicles, respectively. The resulting supernatant was concentrated using a Centricon Plus-70 ultracentrifugal filter device (Merck Millipore, United States) with a molecular weight cutoff of 10 kDa, followed by filtration using a 0.22 μm filter. The concentrated supernatant was then subjected to ultracentrifugation at 100,000 × g for 2 h at 4 °C using a Type 70 Ti rotor (Beckman, United States) to isolate EVs. The sediment was washed with cold PBS and enriched with EVs by centrifugation at 100,000 × g for 2 h at 4 °C. The EV samples were resuspended in PBS and frozen at a −80 °C freezer. Freshly purified EVs were utilized for animal experiments and transcriptomic sequencing.

### 2.3 EVs characterization of TEM

10 μL freshly extracted EV samples was added to a carbon-coated 300-mesh copper grid and dyed with 2% phosphotungstic acid at 37 °C for 3–5 min. Subsequently, the sample was examined by transmission electron microscope (TEM).

### 2.4 EVs characterization of NTA

The size and concentration of EVs were determined by Nanoparticle Tracking Analysis (NTA) using Zetaview-PMX120-Z system (Particle Metrix, Meerbusch, Germany). The EVs samples were diluted in PBS, and the particle concentration was maintained within the optimal detection range to measure the particle size and concentration. NTA measurements were recorded and analyzed at 11 locations. And the temperature was automatically maintained at 22 °C. The zetaview system was calibrated with 110 nm polystyrene particles. The corresponding software ZetaView (version 8.05.14 Sp7) was used for analysis.

### 2.5 EVs labeling and internalization

100 μL of EVs was resuspended in 200 μL Diluent C reagent and then mixed with 4 μL of PKH 26 dye working solution. After incubating the mixture at room temperature for 4 min with periodic pipetting, 500 μL of 5% BSA was added to quench surplus dye. Subsequently, washing them with cold PBS and centrifuged at 100,000 × g for 120 min at 4 °C.

To observe the uptake of EVs, an equal amount of labeled BMSC-derived EVs or PBS was added to BMSCs for 12 h at 37 °C. The BMSCs were fixed in 4% paraformaldehyde at room temperature for 15 min and stained with FITC-labeled phalloidin (Solarbio, China) for cytoskeletal staining and DAPI staining solution (Boster, China) for nuclear staining. Wash the samples with PBS and observe it under a confocal microscope. For quantitative detection of the amount of internalized EVs flow cytometry was performed using DiO-labeled EVs.

### 2.6 Migration assay

5 × 10^4^ BMSCs were seeded into a 12 well plate and incubated overnight at 37 °C. The monolayer of cells was then scratched using the tip of a P200 pipette, followed by two washes with PBS. The culture medium was replaced with low-serum medium. Subsequently, 10 μL of PBS (control), 10 μg/mL EVs from mandible BMSCs (M-BMSCs-EVs) or 10 μg/mL EVs from limb bones BMSCs (L-BMSCs-EVs) were added to the culture medium. After 12 h, images were captured using an inverted microscope (Olympus, Japan) and analyzed using ImageJ software (National Institutes of Health, United States).

### 2.7 Transwell assay and EdU assay

6 × 10^3^ BMSCs were seeded in the upper chamber and the culture medium (without serum) was supplement to 200 μL. And the medium containing 10 μL PBS (control), 10 μg/mL M-BMSCs-EVs or L-BMSCs-EVs was added to the lower chamber (containing 1% exosome-free serum, 600 μL). After incubation at 37 °C for 12 h, the underside cells of the upper chamber were fixed with 4% paraformaldehyde and stained with 10% crystal violet.

BMSCs were seeded on coverslips in 24-well plates at a density of 1 × 10^4^ cells/well and divided into three groups: PBS control, M-BMSCs-EVs treatment, and L-BMSCs-EVs treatment. EdU labeling was performed 12 h later: culture medium and reagent A were mixed at a ratio of 1000:1 to prepare a 50 μM EdU medium, and the cells were incubated for 2 h. After incubation, the cells were washed with PBS, fixed with 4% paraformaldehyde, and permeabilized with 0.5% Triton X-100. Apollo staining solution was then added and incubated at room temperature in the dark for 30 min. DAPI staining was performed, the cells were washed with PBS, and the cells were observed under a confocal microscope.

### 2.8 Osteogenic differentiation induction

BMSCs were planted in a 12-well plate and maintained overnight in MEM-α medium. Osteogenic differentiation was induced using a kit (Cas9x, China) pertaining to the osteogenic differentiation of rat bone marrow mesenchymal stem cells. 10 μL of PBS (control), 10 μg/mL of M-BMSCs-EVs or 10 μg/mL of L-BMSCs-EVs were introduced into the differentiation medium, with the medium being replaced every 2 days.

### 2.9 Immunofluorescence

After 7 days of osteogenic induction, BMSCs were cleaned three times with PBS and fixed with 4% paraformaldehyde for 15 min at room temperature. The cells were subsequently made permeable with 0.1% Triton X-100 for 20 min and blocked with 5% BSA at room temperature for 30 min to avert nonspecific binding. Afterwards, the cells were maintained overnight at 4 °C with primary antibodies targeting against COL I (Servicebio, China) and OCN (Servicebio, China), diluted according to the instructions. After that, the cells were maintained with Alexa Fluor® 488-labeled goat anti-rabbit IgG (Servicebio, China) for 1 h and stained with DAPI for 4 min at the room temperature to make the cell nuclei visible. Cells were observed by a confocal microscope (Leica, Germany).

### 2.10 Alizarin Red staining

After 21 days of osteogenic induction, the cells were cleaned with PBS and fixed with 4% paraformaldehyde for 15 min. Hereafter, the cells were incubated with an Alizarin Red staining solution (Cas9x, China) at room temperature for 5–10 min to evaluate the osteogenic outcome.

### 2.11 Animal experiment

The animal experiment received approval from the Animal Ethics Committee of Shanxi Medical University. All procedures were carried out in line with the guidelines for the care and use of laboratory animals. Altogether 18 male immunodeficient BALB/c nude mice at the age of 6–8 weeks were used for *in vivo* induction of bone tissue formation (n = 6 per group). The mice applied in the study were randomly allotted to the control group or the treatment group. In brief, 5 million third-generation BMSCs were collected and suspended in 100 μL of a matrix gel (ABW, China) containing 1 μg of recombinant bone morphogenetic protein 2 (BMP2, Peprotech, 120-02, United States) to induce bone formation (Li, et al., 2021). Hereafter, 10 μL of PBS (control) or 10 μL of PBS containing 20 μg of freshly purified EVs was mixed with the matrix gel. The cell-loaded matrix gel was then injected under the skin of the immunodeficient mice. Each mouse received one graft. After 4 weeks, the animals were euthanized, and the grafts from each group were gathered for histological characteristics.

### 2.12 Histological evaluation

The samples were promptly fixed in 4% paraformaldehyde for 24 h, dehydrated in a graded series of ethanol, and embedded in paraffin. Specimens were then sectioned (5 μm thickness) and stained with Hematoxylin and Eosin (H&E). Immunohistochemistry and immunofluorescence staining were performed for COL I and OCN. Eventually, images were captured by means of an optical microscope (Olympus, Japan). Semi-quantitative analysis of immunohistochemical and immunofluorescence staining was performed making use of ImageJ software in a blind manner.

### 2.13 Transcriptomic analysis

High-throughput small RNA sequencing was carried out by BGI Genomics Co., Ltd. (BGI Genomics, Shenzhen, China). To put it briefly, the total RNA samples derived from M-BMSCs-EVs (n = 3) and L-BMSCs-EVs (n = 3) were purified through electrophoretic separation on a 15% urea denaturing polyacrylamide gel. The RNA fragments (18-30 nt) were then joined with 5′and 3′adapters, followed by cDNA synthesis employing adapter-specific primers. After PCR amplification, the target fragments ranging from 110 to 130 bp were selected and purified using agarose gel electrophoresis. Sequencing was carried out on the DNBSEQ/MGISEQ-2000 platform. Differentially enriched miRNAs were filtered using the Tom system developed by BGI Genomics. MiRanda was utilized to predict the target genes of the significantly different miRNAs. Gene Ontology (GO) and Kyoto Encyclopedia of Genes and Genomes (KEGG) analyses were carried out on the predicted target genes of the differentially expressed miRNAs.

### 2.14 Statistical analysis

For statistical analysis, SPSS version 22.0 was used. After testing for homogeneity of variances, differences were calculated with the use of one-way analysis of variance (ANOVA) followed by *post hoc* multiple comparison tests. Data were presented as mean ± standard deviation (SD) derived from at least three independent experiments. A P-value <0.05 was considered statistically significant.

## 3 Results

### 3.1 Extraction, characterization, and internalization of EVs

**FIGURE 1:**
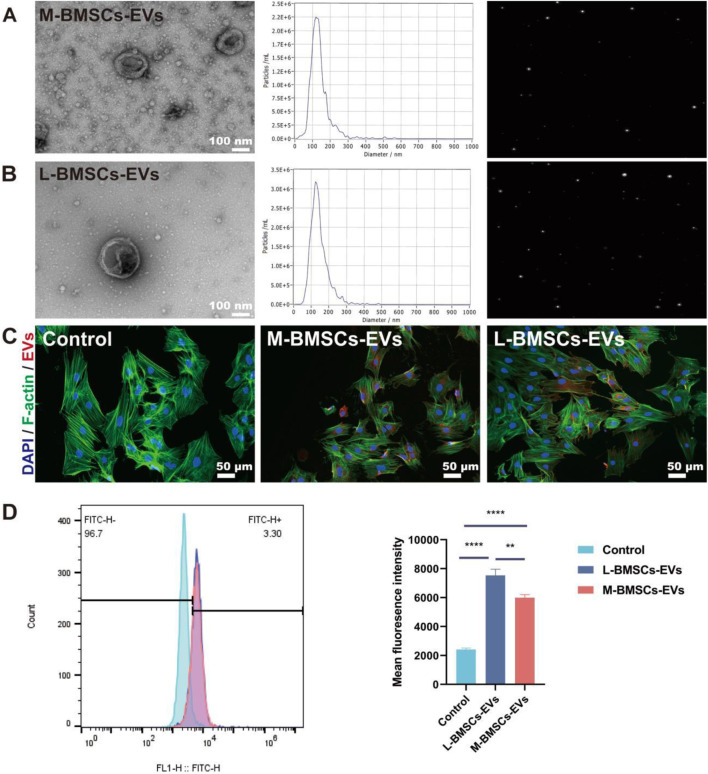
Characterization and internalization of EVs. **(A)** Morphological characterization and NTA of M-BMSCs-EVs. Scale bar: 100 nm. **(B)** Morphological characterization and NTA of L-BMSCs-EVs. Scale bar: 100 nm. **(C)** Internalization of EVs. Scale bar: 50 μm. **(D)** Quantitative analysis of EVs uptake. *p < 0.05, **p < 0.01, ***p < 0.001, ****P < 0.0001, n = 3.

The cell culture supernatants from M-BMSCs and L-BMSCs are gathered and subjected to a series of differential centrifugation procedures to isolate their respective EVs: M-BMSCs-EVs and L-BMSCs-EVs. Subsequently, the EVs are characterized in order to determine potential differences in their physicochemical properties. TEM images revealed that both types of samples exhibited typical cup-shaped vesicles with a bilayer membrane structure, consistent with the morphology of EVs previously reported (Figures 1A,B). NTA showed that M-BMSC-EVs and L-BMSC-EVs exhibited similar size distributions, which is consistent with the TEM results (Figures 1A,B). Furthermore, our results indicated that the average diameters of M-BMSCs-EVs and L-BMSCs-EVs are 123.2 nm and 127.1 nm, respectively, with no significant difference (P > 0.05). This size range is analogous to the size range reported in other studies on MSC-derived EVs. These findings suggest that we successfully extracted EVs derived from BMSCs.

The successful reception of EVs by target cells is a prerequisite for their functional effects. EVs are labeled with PKH 26 (red) and co-incubated with BMSCs for 12 h in order to monitor the delivery of EVs to recipient cells. Confocal microscopy imaging of the cell uptake experiment demonstrated that EVs from both groups are ingested and scattered in the perinuclear region of BMSCs (Figure 1C). Flow cytometry shows that BMSCs take up more L-BMSCs-EVs than M-BMSCs-EVs.

### 3.2 Differential effects of M-BMSCs-EVs and L-BMSCs-EVs on BMSCs migration and proliferation

**FIGURE 2:**
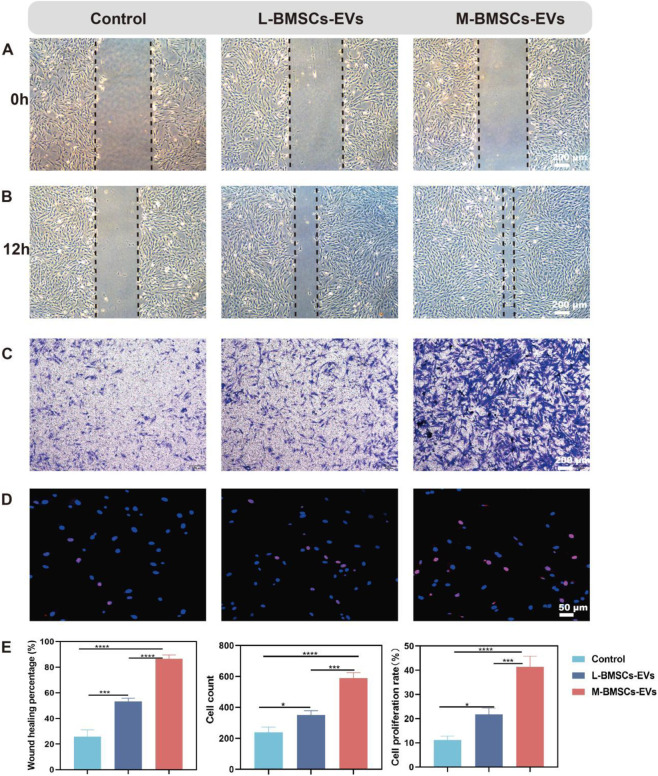
Effects of M-BMSCs-EVs and L-BMSCs-EVs on BMSCs’ physiological functions. **(A,B)** Representative images of the migration assay at 0 h and 12 h. Scale bar: 200 μm. **(C)** Representative images of the Transwell assay at 12 h. Scale bar: 200 μm. **(D)** EdU proliferation assay. Scale bar: 50 μm. **(E)** Quantitative analysis of the migration assay, Transwell assay, and EdU proliferation assay. PBS is used as the control. Data are presented as mean ± SD, *p < 0.05, **p < 0.01, ***p < 0.001, ****P < 0.0001, n = 3.

BMSCs have homing ability, can migrate to the injured site, directly transform into osteoblasts or secrete cytokines and other active substances, and promote osteogenesis and tissue regeneration. Migratory ability is a premise for homing ability. Therefore, they need to maintain vitality, migrate to the injured site, and play a role in promoting tissue regeneration. EVs have been shown to enhance cell viability and migration. We performed a migration assay to validate the effect of different EVs on BMSCs migration. After 12 h, compared with the control group, BMSCs treated with L-BMSCs-EVs or M-BMSCs-EVs showed significantly enhanced migration ability (Figures 2A,B), which increased by about 2.06-fold and 3.35-fold(Figure 2E) respectively.

To further investigate the effect of EVs from different sources on BMSCs’ chemotactic response, Transwell assays are performed to stain with crystal violet after 12 h. Consistent with the previous results, both M-BMSCs-EVs and L-BMSCs-EVs showed superior chemotaxis, with M-BMSCs-EVs had the most significant effect, and there was a marked statistical difference between them (Figures 2C,E).

The EdU assay revealed that both M-BMSCs-EVs and L-BMSCs-EVs promoted BMSCs proliferation, but M-BMSCs-EVs exhibited a more pronounced effect (Figures 2D,E). These results showed that both M-BMSCs-EVs and L-BMSCs-EVs significantly enhance BMSCs’ vitality. At the same concentration, the promoting effect of M-BMSCs-EVs on the physiological function of BMSCs was significantly higher than that of L-BMSCs-EVs.

### 3.3 M-BMSCs-EVs promote osteogenic differentiation of BMSCs more efficiently *in vitro*

**FIGURE 3:**
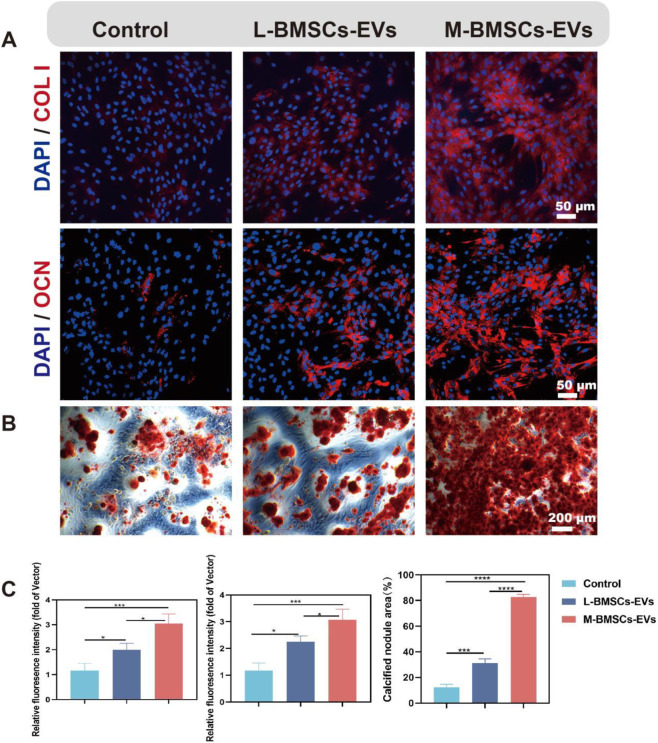
Effects of M-BMSCs-EVs and L-BMSCs-EVs on osteogenic differentiation of BMSCs *in vitro*. **(A)** Immunofluorescence images of COL I and OCN. Scale bar: 50 μm. **(B)** ARS staining of BMSCs after osteogenic induction. Scale bar: 200 μm. **(C)** Quantitative analysis of COL I and OCN immunofluorescence and ARS staining. PBS is used as the control. Data are presented as mean ± SD, *p < 0.05, **p < 0.01, ***p < 0.001, ****P < 0.0001, n = 3.

To assess the impact of different origins of EVs on BMSCs differentiation, immunofluorescence experiments are performed to identify protein expression during osteogenic differentiation. After osteogenic induction, the expression of COL I and OCN proteins in both M-BMSCs-EVs and L-BMSCs-EVs groups is increased significantly compared with the control group (Figures 3A,B). COL I is approximately 1.99-fold and 3.04-fold higher in the L-BMSCs-EVs and M-BMSCs-EVs groups, respectively, compared with the control group (Figure 3C, left). OCN is approximately 2.25-fold and 3.06-fold higher in the L-BMSCs-EVs and M-BMSCs-EVs groups, respectively, compared with the control group (Figure 3C, middle). Compared with the L-BMSCs-EVs group, the COL I expression in the M-BMSCs-EVs group is 1.52 times higher, and the OCN expression is 1.36 times higher.

Furthermore, Alizarin Red S (ARS) staining is performed to further evaluate the osteogenic differentiation of BMSCs. Compared with the control group, the EVs groups showed a significant increase in ARS activity in BMSCs (Figure 3B). BMSCs treated with L-BMSCs-EVs or M-BMSCs-EVs exhibited approximately 2.49-fold and 6.58-fold higher, respectively. M-BMSCs EVs showed the better performance, which is 2.64 times higher than L-BMSCs EVs (Figure 3C, right). Overall, EVs derived from different sources of BMSCs have varying effects on promoting osteogenesis, with M-BMSCs-EVs providing better efficiency.

### 3.4 M-BMSCs-EVs promote osteogenesis more efficiently *in vivo*

To further investigate bone formation *in vivo*, we established a xenograft model by subcutaneously injecting a matrix gel loaded with BMSCs, BMP2, and EVs/PBS into the flank region of nude mice. The BMP2 is used to induce osteogenic microenvironment, and the regenerative capacity of bone tissue is compared between L-BMSCs-EVs and M-BMSCs-EVs. After 4 weeks, the mice are sacrificed, and subcutaneous samples are collected for further evaluation of bone regeneration through histological assessments using H&E staining, immunofluorescence staining, and immunohistochemical staining.

**FIGURE 4:**
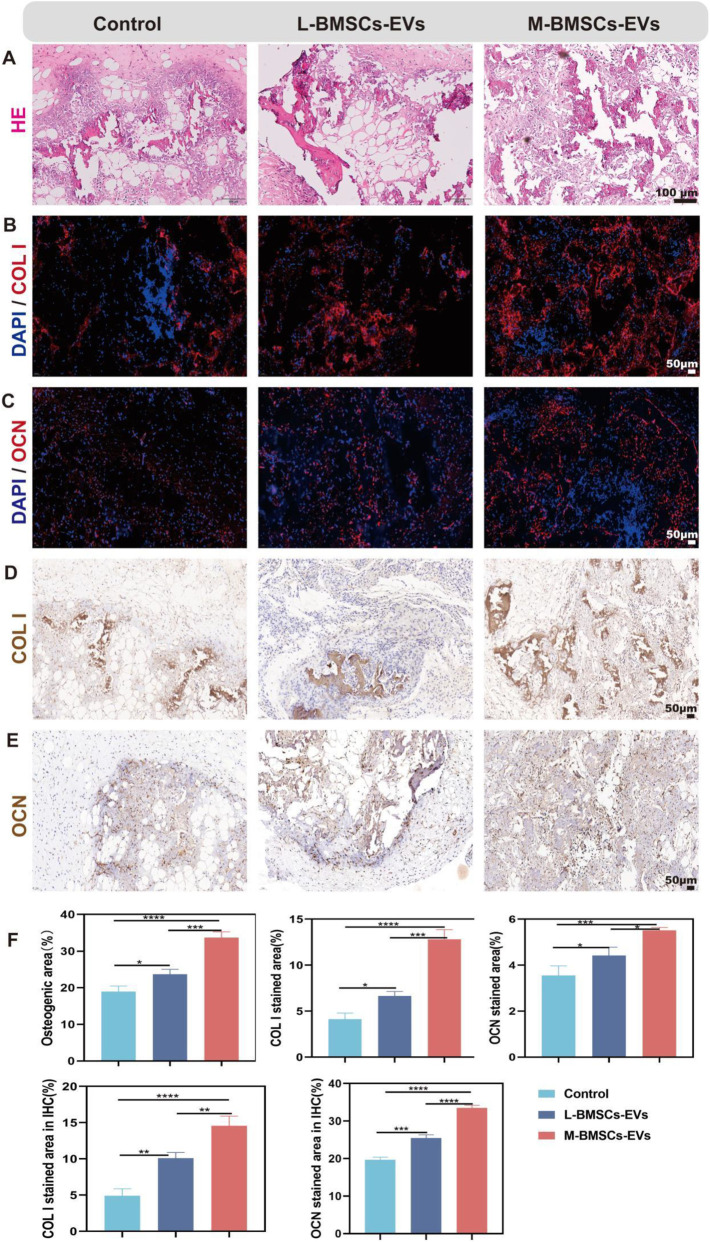
Evaluation of bone regeneration with different EVs *in vivo*. **(A)** H&E staining, Scale bar: 100 μm. **(B,C)** Immunofluorescence staining, Scale bar: 50 μm, and **(D,E)** Immunohistochemical staining, Scale bar: 50 μm.

H&E staining revealed the formation of newly generated bone-like tissue in all three groups (Figure 4A), with more bone like tissue in L-BMSCs-EVs and M-BMSCs-EVs, indicating enhanced bone formation induced by EVs. Among the groups, the M-BMSCs-EVs group exhibited the most abundant bone-like tissue (Figure 4F). Immunofluorescence and immunohistochemical staining are then performed to analyze the staining of bone-specific proteins COL I and OCN in the newly formed tissue. Immunofluorescence staining showed increased expression of COL I and OCN proteins in both L-BMSCs-EVs and M-BMSCs-EVs groups compared with the control group, with the M-BMSCs-EVs group showing the highest expression (Figures 4B,C,F). Immunohistochemical staining demonstrated strong positive staining for COL I and OCN proteins in the L-BMSCs-EVs and M-BMSCs-EVs groups, whereas relatively weak staining is observed in the control group, indicating a visible contribution of EVs derived from BMSCs to the process of bone regeneration. Additionally, the M-BMSCs-EVs group exhibited significantly higher expression of COL I and OCN proteins, indicating that M-BMSCs-EVs demonstrated a more efficient promotion of bone regeneration among all the groups (Figures 4D–F).

These findings are consistent with previous discoveries, suggesting the positive role of EVs in bone regeneration, and further highlighting the higher osteogenic potential of M-BMSCs-EVs.

### 3.5 The differential miRNA profile of M-BMSCs-EVs and L-BMSCs-EVs could be a potential mechanism

**FIGURE 5:**
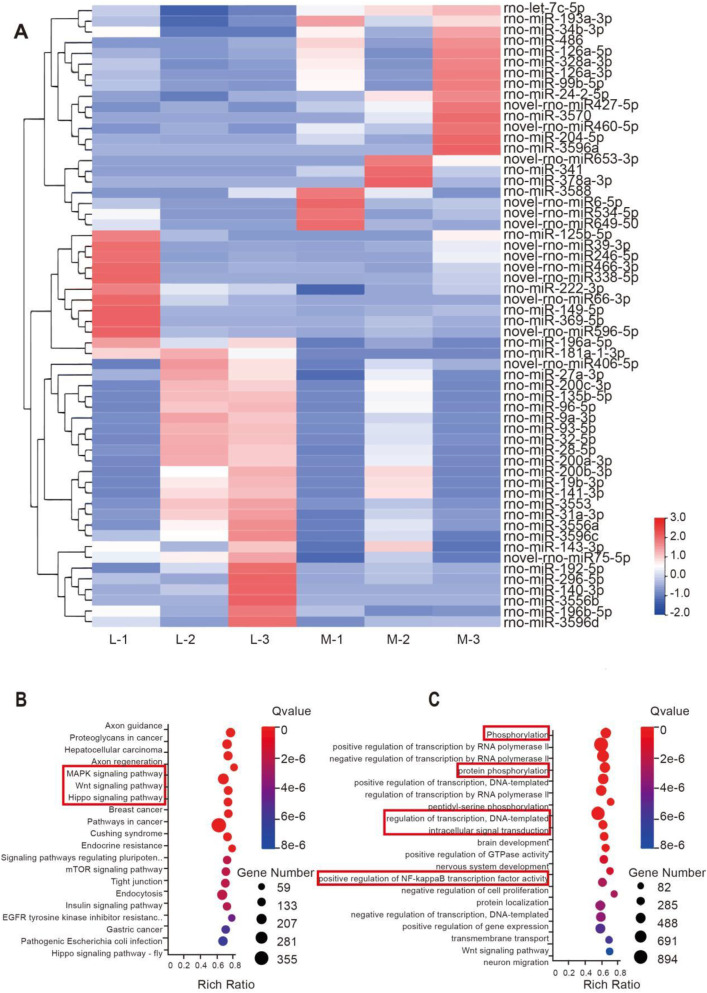
Transcriptional profiling of EVs reveals potential mechanisms underlying the differential impact of EVs. **(A)** Heatmap displaying the distinct miRNA profiles of M-BMSCs-EVs and L-BMSCs-EVs. **(B)** KEGG enrichment analysis of the upregulated miRNAs’ predicted target genes. **(C)** GO enrichment analysis of the upregulated miRNAs’ predicted target genes.

miRNAs encapsulated in EVs play a crucial role in intercellular communication. To explore the potential biological mechanisms underlying the differential effects of EVs derived from different sources on bone tissue regeneration, we conducted transcriptomic profiling to compare M-BMSCs-EVs and L-BMSCs-EVs at the miRNA level. Only miRNAs showing significant upregulation or downregulation with an average fold change of 1.2 are selected for further analysis. As expected, the two types of EVs exhibited distinct miRNA profiles, indicating that the source influences the characteristics of EVs (Figure 5A). Compared with L-BMSCs-EVs, M-BMSCs-EVs showed 21 significantly upregulated differentially expressed miRNAs (Figure 5A). Enrichment analysis of the predicted target mRNA of these differentially expressed miRNAs is further performed.

Gene Ontology (GO) enrichment analysis revealed significant enrichment of processes involved in protein phosphorylation, transcriptional regulation, intracellular signaling, and positive regulation of NF-κB transcription factor activity in M-BMSCs-EVs (Figure 5C). Phosphorylation, cellular transcriptional regulation, and intracellular signaling are all involved in bone regeneration and osteogenic differentiation. Furthermore, studies have shown that TNF-α promotes osteogenic differentiation of human mesenchymal stem cells by triggering the NF-κB signaling pathway (Hess, et al., 2009). Kyoto Encyclopedia of Genes and Genomes (KEGG) pathway analysis revealed significant upregulation of the MAPK signaling pathway, Wnt signaling pathway, and Hippo signaling pathway in the comparison (Figure 5B). This suggests that mRNA associated with these pathways has a higher abundance in M-BMSCs-EVs compared with L-BMSCs-EVs, which may contribute to enhanced bone tissue regeneration.

## 4 Discussion

We isolated and identified M-BMSCs-EVs and L-BMSCs-EVs. Both EVs can be internalized by BMSCs, and can promote the proliferation and migration of BMSCs. *In vitro* and *in vivo* experiments, M-BMSCs-EVs showed better performance in promoting osteogenic differentiation and bone regeneration. The possible mechanisms of these differences were identified by transcriptional profiling. M-BMSCs-EVs and L-BMSCs-EVs have different miRNA profiles. The target genes predicted by upregulated miRNA are enriched in key signaling pathways such as MAPK, Wnt and Hippo.

Melatonin enhances osteogenic differentiation of dental pulp stem cells and promotes cranial bone defect regeneration efficiency by regulating the MAPK pathway (Chan, et al., 2022). Bone-targeting miR-26a-loaded exosome mimetics promote bone regeneration therapy by activating the Wnt signaling pathway (Sun, et al., 2019). Long non-coding RNA ZBED3-AS1 inhibits IL-1β-induced mesenchymal stem cell differentiation and enhances bone regeneration through the Wnt/β-catenin signaling pathway (Hu, et al., 2019). Ap-2β regulates cranial bone osteogenic potential through the activation of the Wnt/β-catenin signaling pathway (Hu, et al., 2023). Studies have shown that the Hippo/LATS1/YAP1 axis promotes bone regeneration during tension-induced osteogenesis through the activation of the Wnt/β-catenin pathway (Kehan, et al., 2023).

It is important to acknowledge the significant species-specific differences that exist between rodents and humans. Subcutaneous transplantation model does not replicate the complex physiological and biomechanical milieu of an orthotopic bone defect site. Future studies need to use bone defect model to assess the therapeutic efficacy of M-BMSC-EVs. Additionally, while transcriptional profiling analysis has provided important clues, it alone may not fully elucidate the intricate underlying mechanisms of EVs. Future studies will combine pharmacological inhibition and gene knockout to clarify the direct regulatory mechanisms of EVs-miRNA on these pathways.

## 5 Conclusion

M-BMSCs-EVs may be an excellent candidate for improving bone healing in autologous bone transplantation, bone tissue engineering, or other bone diseases.

## Data Availability

The datasets presented in this study can be found in online repositories. The names of the repository/repositories and accession number(s) can be found in the article/supplementary material.
